# Inter-pregnancy interval and associated adverse maternal outcomes among women who delivered at Kilimanjaro Christian Medical Centre in Tanzania, 2000-2015

**DOI:** 10.1371/journal.pone.0228330

**Published:** 2020-02-06

**Authors:** Leah Anku Sanga, Tara Mtuy, Rune Nathaniel Philemon, Michael Johnson Mahande

**Affiliations:** 1 Department of Epidemiology & Biostatistics, Institute of Public Health, Kilimanjaro Christian Medical University College, Kilimanjaro, Tanzania; 2 Department of Global Health & Development, London School of Hygiene & Tropical Medicine, London, United Kingdom; 3 Department of Community Health, Institute of Public Health, Kilimanjaro Christian Medical University College, Kilimanjaro, Tanzania; University of Cape Coast, GHANA

## Abstract

Inter-pregnancy interval is an important determinant of both maternal and child health. Suboptimal inter-pregnancy interval has been associated with adverse maternal outcomes -including postpartum hemorrhage and hypertensive disorders, direct causes of maternal mortality. Both overall maternal mortality and the contribution of postpartum hemorrhage on maternal mortality have increased in Tanzania. If we are to achieve sustainable development goal (SDG) number 3.1 to reduce the global maternal mortality ration to less than 70 per 100,000 live births by 2030, it is highly important that such contributors are dealt with. This study aimed to determine the distribution and trends of inter-pregnancy interval and to deduce its association with adverse maternal outcomes among women who delivered at Kilimanjaro Christian Medical Centre (2000–2015).A retrospective cohort study was designed using Kilimanjaro Christian Medical Centre medical birth registry data for women who delivered from 2000 to 2015. Women with at least two births recorded in the registry were included. A total of 7,995 births from 6,612 mothers were analyzed. Anemia during pregnancy, post-partum hemorrhage and pre-eclampsia were adverse maternal outcomes of interest. Data analysis was performed using multivariable logistic regression models allowing for robust standard errors. Crude and adjusted odds ratio with their respective 95% confidence intervals were estimated. More than half (51.7%) of non-first births were born within sub-optimal IPI. The median IPI was 34 months (IQR: 33.5 months). The median IPI increased from 11 months in 2002 to 35 months in 2006 and plateaued until 2014, but it rose to 41.6 months in 2015. Median IPI was shorter in young women <20 years and in birth order seven and above (16 vs. 27 months, respectively). Short IPI was associated with lower risk of pre-eclampsia [aOR: 0.71, 95%CI: 0.52, 0.97] while long IPI was associated with lower risk of post-partum hemorrhage [aOR: 0.70, 95%CI: 0.52, 0.94]. This study found an association between long and short IPI with adverse maternal outcomes. Even though these results should be interpreted with caution based on the fact that the data was sampled from a referral hospital and hence there could be overrepresentation of women with maternal complications, our findings still warrant the importance of supporting modern family planning methods as a measure to improve IPI and thereby improve maternal outcomes as well.

## Introduction

Inter-pregnancy interval (IPI) is the time lapse between termination of one pregnancy and conception of a subsequent one[1]. Optimal IPI can ensure optimal health for both mother and infant [2] whereas sub-optimal IPI has been associated with several maternal morbidities and mortality [3–10]. World Health Organization (WHO) recommends an interval of at least 24 months between a live birth and the next pregnancy [1] while others have argued that IPI of 3–5 years further reduces the risk for adverse maternal outcomes [11]. Different theories—particularly maternal depletion theory and physiological regression theory—link inter-pregnancy interval with adverse maternal outcomes [3,12].

**Fig 1:**
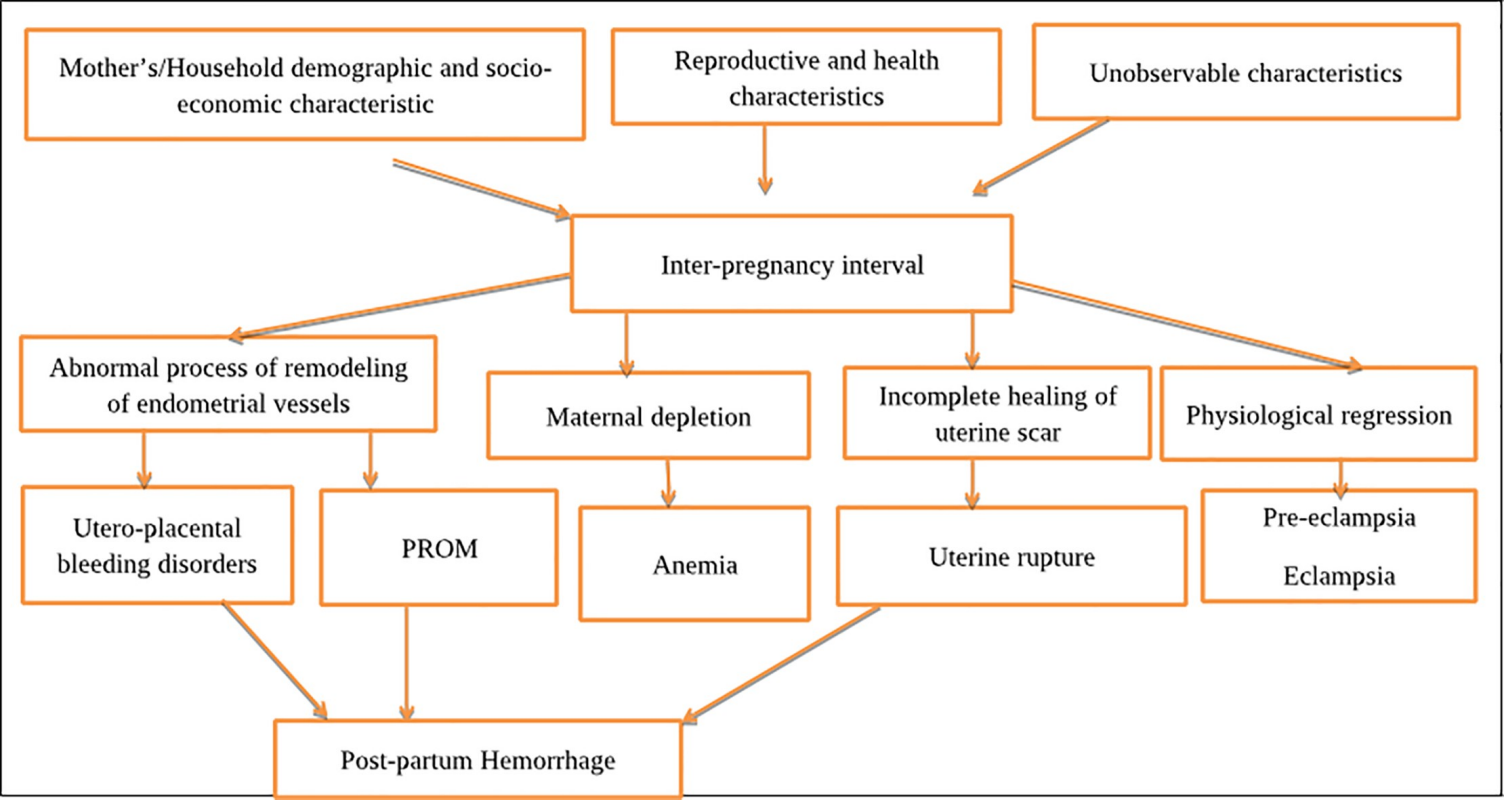
Directed acyclic diagram showing relationship between inter-pregnancy interval and adverse maternal outcomes.

Hemorrhage and hypertensive disorders are among maternal morbidities associated with IPI (Fig **1**). They are among direct causes of maternal deaths and account for approximately half of the maternal deaths globally [13–15]. Approximately 800 women die every day globally due to pregnancy and childbirth related complications [14] with the global maternal mortality ratio (MMR) being 211 per 100,000 live births[16]. Most (99%) of these deaths occur in low income countries, with majority (66%) from Sub-Saharan Africa, including Tanzania [14,17]. It is estimated that 20–35% of maternal deaths can be prevented by adhering to recommended IPI [18].

Maternal mortality and morbidity can be averted by dealing with preventable factors that cause them. Despite several interventions[19–22], maternal mortality has risen from 432 in 2012 to 524 per 100,000 live births in 2017 in Tanzania[16]. The country failed to reach the Millennium Development Goal (MDG) number 5 target for improved maternal health and the trend is not promising to enhance achievement of Sustainable Development Goal number 3 to reduce maternal mortality ratio to less than 70 per 100,000 live births by 2030 [20,23,24]. Furthermore, the proportion of maternal deaths attributed to hemorrhage has increased from 15% in 2010 to 18% in 2015 and there has been no improvement in proportion of deaths caused by hypertensive disorders from 2006 to 2015 [19,25].

Several factors have been reported to influence IPI [26–30], but of ultimate importance is modern contraceptive use[30]. However, utilization of modern contraceptives in Tanzania is still low and the proportion of births born within short IPI has increased by 3% in the past decade [19,25].

Non-adherence to optimal IPI could be one of the drivers increasing MMR in Tanzania since it is associated with direct causes of maternal mortality. However, in our settings, little is known about the role of IPI on adverse maternal outcomes. Understanding the association between IPI and adverse maternal outcomes may help to provide important information needed to help design evidence-based interventions to reduce its impact, and hence accelerate the achievement of SDG3 by 2030. This study aimed to determine the distribution, trend and patterns of IPI among women who delivered at Kilimanjaro Christian Medical Centre in northern Tanzania between 2005 and 2015. The study also assessed the association between IPI and adverse maternal outcomes.

## Materials and methods

### Study setting

This study was conducted at the Department of Obstetrics and Gynecology of Kilimanjaro Christian Medical Centre (KCMC). KCMC is a consultant and university teaching hospital located in Moshi urban district, Kilimanjaro region in northern Tanzania. It is one of the four zonal hospitals in Tanzania which serves over 15 million people, including the local community and referred cases from six regions in Tanzania. Pregnant high-risk women are referred to KCMC for observation and delivery while women from the local community may come to deliver on their own accord. The main catchment area of KCMC hospital is Kilimanjaro region.

### Design, sample and sampling

This study used secondary data from a cohort of women who delivered at KCMC from 2000 to 2015. Women were followed retrospectively through their medical records. We restricted our analysis to two or more deliveries from the same mother, delivered at KCMC during the study period. Women who did not have consecutive deliveries during the study time were excluded. A total of 19,000 deliveries were recorded during the study period. Of these, 7,995 births from 6,612 mothers were eligible for the study.

### Data collection

Data were extracted from the medical birth registry at KCMC. The medical birth registry records information for all mothers who deliver in the Obstetrics and Gynecology Department at KCMC within 24 hours after delivery or as soon as mothers have recovered in case of complicated deliveries. Information was collected on a daily basis by a trained nurse/midwife including, but not limited to, maternal and paternal socio-demographics, mother’s reproductive history and conditions and complications of the mother before, during and after pregnancy/delivery and puerperium. In the interview, women also provided information about births delivered outside KCMC during the study period. However, information on miscarriages and abortions, whether they happened at KCMC or elsewhere, were not captured in the birth registry. Each woman and her pregnancy outcome were given a unique identifier which was used to link deliveries to a specific mother. Data were entered in a computerized system of the birth registry.

### Data analysis

Data analysis was performed using STATA version 13.1 statistical software (StatCorp, College Station, TX).

The study looked at three adverse maternal outcomes; post-partum hemorrhage (PPH), pre-eclampsia and anemia. PPH was considered if a woman lost ≥500mls of blood after vaginal delivery or ≥1000mls of blood after caesarean delivery [1]. Blood loss was visually estimated by a nurse midwife after vaginal delivery and by the doctor performing the surgery in caesarean delivery. A woman was considered to have anemia if at any point in her pregnancy she had measured (by any validated method) Hemoglobin level below 8.5 grams per deciliter (8.5g/dl). Pre-eclampsia was defined as a disorder characterized by development of hypertension 140/90 mm Hg or more with proteinuria after the 20^th^ week of gestation in a previously normotensive and non-proteinuric woman[1].

Inter-pregnancy interval (IPI) was the main exposure for these outcomes and it was computed as follows:

IPI(days)=[D.O.Bofindexchild−D.O.Bofprecedingchild]−Gestationageatbirthofindexchild

IPI was categorized into <24 months (short IPI), 24–59 (reference group/optimal IPI) and 60+ months (long IPI). Mother’s age, education level, occupation, religion, marital status, tribe, current residence, pregnancy type, family planning use, alcohol use, anemia in previous pregnancy, PPH in previous pregnancy, pre-eclampsia in previous pregnancy, referral status, delivery mode, parity, any ANC visit, number of ANC visits and death of a preceding child were considered as confounders.

Since children are clustered within a mother, hierarchy of data was considered but there was no effect of clustering on the outcomes. In the univariate and multivariable analysis, logistic regression with robust standard errors was used to assess the association between IPI and each of the selected adverse maternal outcomes. Confounding was established if a variable changed the Odds Ratios for the effect of IPI on any adverse maternal outcome by 10% or more. To determine if there was a dose-response relationship between IPI and adverse maternal outcomes, Generalized Additive Models were used. However, all three maternal outcomes were deemed to have a linear association with IPI. While analyzing trends of IPI over the years, the years 2000 and 2001 were excluded from the analysis due to few numbers of subjects. Significance was considered at 5% level.

### Ethical considerations

For practical reasons, since the interview was administered just after the woman had given birth, consent was given orally. The midwife nurse gave every woman oral information about the birth registry, the data needed to be collected from them and the use of the data for research purposes. Then informed consent was sought verbally from every woman. Following the consent, the woman could still opt not to reply to individual questions. Inclusion of the orally conducted interview in the database was conditional on informed consent which was followed as far as the orally conducted interview was concerned. All consent procedures were approved by the Kilimanjaro Christian Medical Centre ethical committee.

Ethical clearance for the establishment of Medical Birth registry was granted from Ethics Committee at Kilimanjaro Christian Medical Centre, approved by Tanzania’s Ministry of Health and National Ethics Committee in Norway. Approval to carry out this study was obtained from Tumaini University Makumira through Kilimanjaro Christian Medical University College Research and Ethical Committee.

## Results

### Socio-demographic and pregnancy characteristics of study participants at baseline delivery

The mean mother’s age of all women in the sample was 30.5 (SD: 5.3 years) with most women aged between 20–34 years. The majority of women were employed, married and living in urban areas (Table **1**). Most of them used modern family planning and attended four or more ANC visits. A lower proportion (2.8%) of women with long IPI had PPH in preceding pregnancy while a higher proportion of women with short IPI had pre-eclampsia (4.9%) and had experienced perinatal death in their preceding birth (11.2%) (Table 2).

**Table 1:** Socio-demographic characteristics of women by inter-pregnancy interval at baseline (N = 6,612).

	Inter-pregnancy interval (months)					
	<24 N = 2,174		24–59 N = 3,193		60+ N = 1,245	
Variable	n	%	n	%	n	%
Age categories						
≤19	51	2.3	11	0.3	3	0.2
20–34	1,853	85.2	2,581	80.8	801	64.3
≥35	268	12.3	596	18.7	441	35.4
Missing	2	0.1	5	0.2	-	-
Mother's education						
No formal education	28	1.3	29	0.9	13	1.0
Primary	1,075	49.4	1,750	54.8	720	57.8
Secondary and above	1,068	49.1	1,409	44.1	512	41.1
Missing	3	0.1	5	0.2	-	-
Mother's occupation						
Employed	1,528	70.3	2,370	74.2	1,010	81.1
Unemployed	636	29.3	814	25.5	235	18.9
Missing	10	0.5	9	0.3	-	-
Religion						
Catholic	911	41.9	1,243	38.9	521	41.8
Protestant	852	39.2	1,314	41.2	491	39.4
Muslim	398	18.3	621	19.4	229	18.4
Others	7	0.3	11	0.3	2	0.2
Missing	6	0.3	4	0.1	2	0.2
Marital status						
Married	2,094	96.3	3,098	97.0	1,182	94.9
Single	72	3.3	83	2.6	63	5.1
Missing	8	0.4	12	0.4	-	-
Mother's current residence						
Rural	764	35.1	1,055	33	366	29.4
Urban	1,301	59.8	2,018	63.2	827	66.4
Semi urban	107	4.9	119	3.7	52	4.2
Missing	2	0.1	1	<0.01	-	-

Modern family planning include: pills, injections, implants and intra-uterine devices

**Table 2:** Health-related characteristics of women by inter-pregnancy interval at baseline birth (N = 6,612).

	Inter-pregnancy interval (Months)					
	<24 N = 2,174		24–59 N = 3,193		60+ N = 1,245	
VARIABLES	n	%	n	%	n	%
**Type of family planning used**						
** **None	375	17.2	179	5.6	61	4.9
** **Modern	1,073	49.4	2,335	73.1	1,034	83.1
** **Traditional	708	32.6	660	20.7	147	11.8
** **Missing	18	0.8	19	0.6	3	0.2
**Alcohol use before/during pregnancy**	587	27.0	984	30.8	430	34.5
**PPH in preceding birth**	74	3.4	112	3.5	35	2.8
**Anemia in preceding birth**	36	1.7	53	1.7	15	1.2
**Preeclampsia in preceding birth**	106	4.9	110	3.4	43	3.5
**Referred from**						
** **Home	1,945	89.5	2,964	92.8	1,183	95.0
** **Regional hospital	44	2.0	73	2.3	19	1.5
** **District hospital	34	1.6	42	1.3	19	1.5
** **Other	44	2.0	71	2.2	23	1.8
** **Missing	107	4.9	43	1.3	1	0.1
**Delivery mode**						
** **Spontaneous	1,442	66.3	1,943	60.9	726	58.3
** **Assisted	19	0.9	36	1.1	16	1.3
** **CS	710	32.7	1,208	37.8	502	40.3
** **Missing	3	0.1	6	0.2	1	0.1
**Death of preceding birth**	243	11.2	77	2.4	17	1.4
** **Missing	4	0.2	10	0.3	2	0.2
**Parity**						
** **2	1,301	59.8	1,765	55.3	550	44.2
** **3	490	22.5	834	26.1	412	33.1
** **>3	383	17.6	594	18.6	283	22.7
**Antenatal care attendance**	2,162	99.6	3,178	99.8	1,241	99.7
**Number of ANC visits**						
** **<4	864	39.7	1,082	33.9	442	35.5
** **≥4	1,279	58.8	2,083	65.2	794	63.8
** **Missing	31	1.4	28	0.9	9	0.7

### Characteristics of women who had adverse maternal outcomes

Of all pregnancies studied, a total of 309 (3.9%) experienced PPH, 80 (1.0%) experienced anaemia and 270 (3.4%) experienced Pre-eclampsia. Distribution of proportion of these outcomes by inter-pregnancy interval is explained in Table **3**. A total of 692 (10.5%) women experienced one of the adverse maternal outcomes in at least one of their subsequent deliveries. PPH was the most frequent adverse outcome (53%) among women who had adverse maternal outcomes. A few (12%) of these women had anemia and 39% had pre-eclampsia.

**Table 3:** Proportion of women with adverse outcomes by inter-pregnancy interval (N = 7,995).

	Total	PPH (N = 369)		ANAEMIA (N = 80)		PRE-ECLAMPSIA (N = 270)	
IPI categories (months)	N	n	%	n	%	n	%
24–59	3,890	205	5.3	47	1.2	138	3.5
<24	2,625	100	3.8	26	1.0	77	2.9
>59	1,480	64	4.3	7	0.5	55	3.7

More than half of all women in the sample had IPI of 24–59 months (51.1%), were aged 20-34years (65.9%) and were married (97%) (Table **4**).

**Table 4:** Socio-demographic characteristics of women by adverse maternal outcomes (N = 692).

	PPH (n = 369)		Anemia (n = 80)		Pre-eclampsia(n = 270)	
VARIABLES	n	%	n	%	n	%
**IPI categories**						
** **24–59	205	55.6	47	58.8	138	51.1
** **<24	100	27.1	26	32.5	77	28.5
** **>59	64	17.3	7	8.8	55	20.4
**Age categories**						
** **≤19	1	0.3	-	-	-	-
** **20–34	270	73.2	63	78.8	178	65.9
** **≥35	98	26.6	17	21.3	92	34.1
**Education**						
** **None	4	1.1	-	-	1	0.4
** **Primary	206	56.1	40	50.0	112	41.5
** **Secondary and above	157	42.8	40	50.0	157	58.1
**Occupation**						
** **Employed	273	74.2	49	61.3	217	80.4
** **Unemployed	95	25.8	31	38.8	53	19.6
**Religion**						
** **Catholic	144	39.1	29	36.3	82	30.4
** **Protestant	151	41	36	45	124	45.9
** **Muslim	72	19.6	15	18.8	64	23.7
** **Others	1	0.3	-	-	-	-
**Marital status**						
** **Married	352	96.4	75	94.9	261	97
** **Single	13	3.6	4	5.1	8	3
**Current residence**						
** **Rural	142	38.5	29	36.3	69	25.6
** **Urban	209	56.6	45	56.3	188	69.6
** **Semi urban	18	4.9	6	7.5	13	4.8

Across all adverse outcomes, most women delivered by caesarean section. Higher proportion (14.1%) of women who had pre-eclampsia had experienced perinatal death in the preceding child compared to those who had anemia (3.8%) and PPH (8.4%)(Table **5**).

**Table 5:** Health-related characteristics of women by adverse maternal outcomes (N = 692).

	PPH (n = 369)		Anemia (n = 80)		Pre-eclampsia (n = 270)	
Variable	n	%	n	%	n	%
**Type of family planning used**						
** **None	33	9.1	10	12.5	25	9.4
** **Modern	259	71.2	45	56.3	165	61.8
** **Traditional	72	19.8	25	31.3	77	28.8
**Alcohol use before/during pregnancy**	108	29.3	24	30	67	24.8
**PPH in preceding birth**	22	6	1	1.3	13	4.8
**Anemia in preceding birth**	9	2.4	6	7.5	5	1.9
**Preeclampsia in preceding birth**	16	4.3	5	6.3	70	25.9
**Referred from**						
** **Home	338	91.6	75	94.9	237	90.1
** **Regional hospital	9	2.4	-	-	9	3.4
** **District hospital	9	2.4	2	2.5	7	2.7
** **Other	13	3.5	2	2.5	10	3.8
**Delivery mode**						
** **Spontaneous	50	13.6	39	48.8	115	42.8
** **Assisted	2	0.5	1	1.3	7	2.6
** **CS	317	85.9	40	50.0	147	54.6
**Death of preceding birth**	31	8.4	3	3.8	38	14.1
**Parity**						
** **2	135	36.6	43	53.8	86	31.9
** **3	120	32.5	14	17.5	90	33.3
** **>3	114	30.9	23	28.7	94	34.8
**Antenatal care attendance**	367	99.5	80	100.0	269	99.6
**Number of ANC visits**						
** **<4	133	36.0	27	33.8	97	35.9
** **≥4	231	62.6	51	63.8	169	62.6
** **Missing	5	1.4	2	2.5	4	1.5

### Distribution and trends of IPI across birth orders

**Fig 2:**
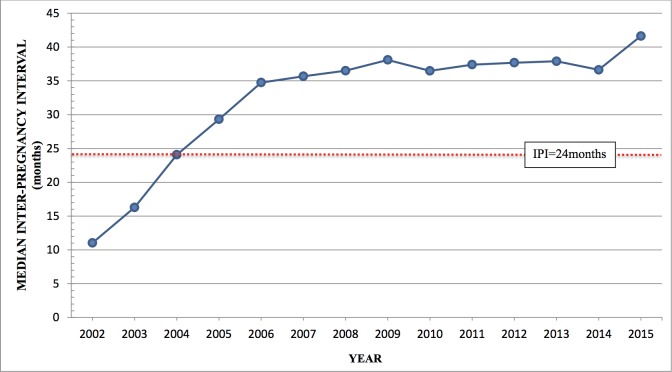
Trend of inter-pregnancy interval over time. The red dotted line shows a demarcation for IPI at 24 and 59 months.

About half of the women had sub-optimal IPI (short = 32.9%; long = 18.8%) with the median IPI 34months (IQR: 33.4months). The median IPI improved from 11 months in 2002 to 34.8 months in 2006 and remained stable up to 2014. In 2015, it raised to 41.6 months (Fig **2**).

**Fig 3:**
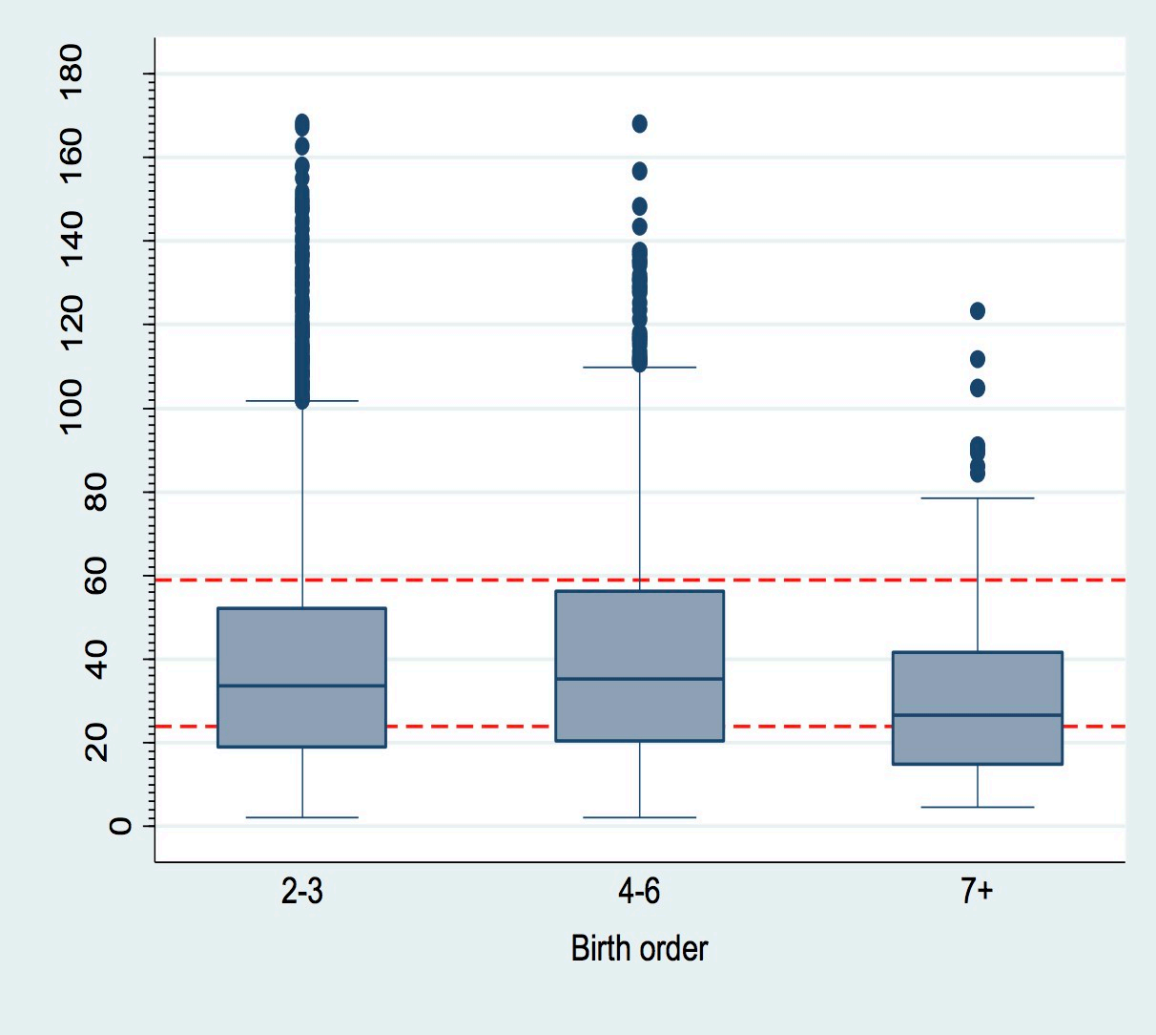
Distribution of inter-pregnancy interval by birth order. The red dotted line shows a demarcation for IPI at 24 and 59 months.

There was no difference in median IPI in the 2^nd^ to 3^rd^ birth order to that in 4^th^ to 6^th^ birth order (median IPI = 33.6 and 35 months, respectively). In higher birth orders (7 and above), median IPI was shorter (26.6 months) compared to lower birth orders (Fig **3**).

**Fig 4:**
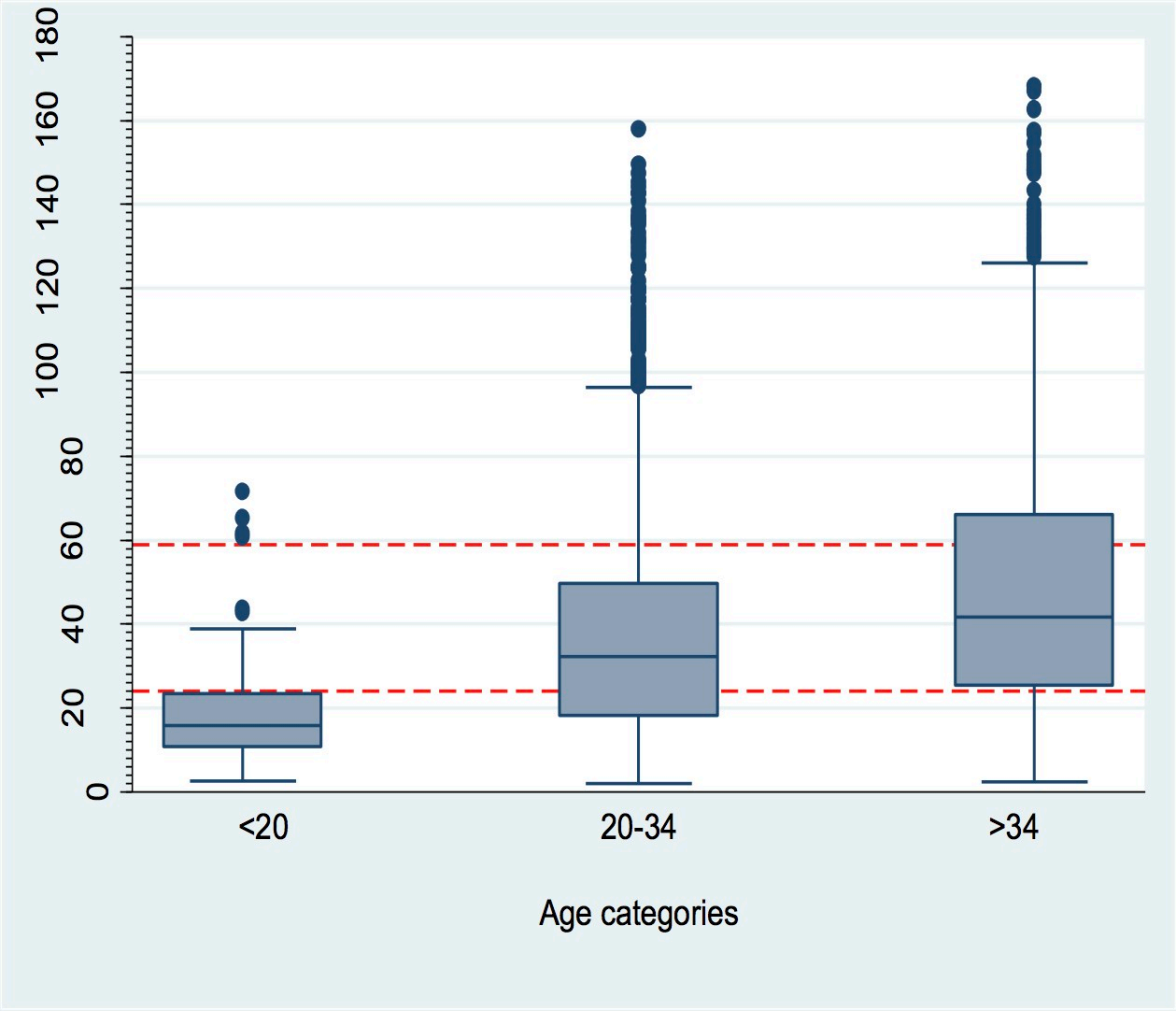
Distribution of inter-pregnancy by age-group. The red dotted line shows a demarcation for IPI at 24 and 59 months.

The median IPI was longer in older women aged 35 and above (median IPI = 32.3 months) and shorter in younger women <20 years (median IPI = 15.7months) (Fig **4**).

### Association between IPI and adverse maternal outcomes

In the univariate analysis, long IPI was significantly associated with anemia at 5% level while there was no significant association between long IPI and pre-eclampsia or PPH. Short IPI was not significantly associated with any of the adverse maternal outcomes at 5% level. Parity, delivery mode and pregnancy type were associated with all of the three adverse maternal outcomes in univariate analysis (Table **6**). After adjusting for other variables, short IPI was significantly associated with lower risk of pre-eclampsia while long IPI was significantly associated with lower risk of PPH at 5% level. Women with short IPI had 29% lower odds of having pre-eclampsia as compared to those with optimal IPI [aOR: 0.71, 95%CI: 0.52, 0.97]. On the other hand, women with long IPI had 30% lower odds for post-partum hemorrhage [aOR: 0.70, 95%CI: 0.52, 0.94] compared to those with optimal IPI (Table **7**).

**Table 6:** Crude association between inter-pregnancy interval and other confounders with adverse maternal outcomes.

	PPH (N = 7,360)		ANAEMIA (N = 7,995)		PRE-ECLAMPSIA (N = 7,995)	
VARIABLE	cOR	P-value	cOR	P-value	cOR	P-value
**IPI Categories**						
24–59	1.00		1.00		1.00	
<24	0.82	0.12	0.82	0.407	0.82	0.174
>59	0.77	0.081	0.39	**0.02**	1.05	0.767
**Age (yrs)**						
<20	1.00					
20–34	2.49	0.368				
>34	2.97	0.284				
**Mother's education**						
No formal education	1.00				1.00	
Primary	0.91	0.851			2.11	0.461
Secondary and above	0.76	0.601			3.51	0.214
**Mother's occupation**						
Employed	1.00		1.00		1.00	
Unemployed	1.13	0.302	1.93	**0.005**	0.73	**0.05**
**Marital status**						
Married	1.00		1.00		1.00	
Single	1.26	0.421	1.78	0.265	1.01	0.969
**Mother's current residence**						
Rural	1.00		1.00		1.00	
Urban	0.71	**0.003**	0.80	0.36	1.42	**0.019**
Semi urban	1.02	0.942	1.64	0.275	1.51	0.236
**Type of pregnancy**						
Multiple	1.00		1.00		1.00	
Singleton	0.35	**<0.001**	0.29	**0.01**	0.46	**0.014**
**Type of family planning used**						
None	1.00		1.00		1.00	
Modern	0.91	0.603	0.55	0.088	0.80	0.323
Traditional	0.78	0.259	0.90	0.785	1.11	0.632
**Alcohol use before/during pregnancy**						
No	1.00		1.00		1.00	
Yes	1.05	0.673	1.01	0.96	0.77	**0.08**
**PPH in preceding birth**						
No	1.00		1.00		1.00	
Yes	1.62	**0.034**	0.34	0.277	1.38	0.269
**Anemia in preceding birth**						
No	1.00		1.00		1.00	
Yes	1.64	0.156	5.27	**0.001**	1.19	0.712
**Preeclampsia in preceding birth**						
No	1.00		1.00		1.00	
Yes	1.24	0.409	1.73	0.238	11.50	**<0.0001**
**Referred from**						
Home	1.00				1.00	
Regional hospital	1.59	0.185			1.98	0.052
District hospital	2.03	**0.045**			2.14	0.055
Other	1.89	**0.031**			2.09	0.027
**Delivery mode**						
Spontaneous	1.00		1.00		1.00	
Assisted	2.47	0.215	1.45	0.7	3.72	**0.001**
CS	11.98	**<0.001**	1.73	**0.017**	2.20	**<0.0001**
**Death of preceding birth**						
No	1.00		1.00		1.00	
Yes	2.16	**<0.001**	0.78	0.679	3.50	**<0.0001**
**Parity**						** **
2	1.00		1.00		1.00	** **
3	1.29	**0.047**	0.47	**0.013**	1.54	**0.003**
>3	1.67	**<0.001**	1.02	0.923	2.15	**<0.0001**
**Antenatal care attendance**						
No					1.00	
Yes					1.14	0.896
**Number of ANC visits**						
<4	1.00		1.00		1.00	
≥4	1.08	0.505	1.13	0.603	1.04	0.784

cOR = Crude Odds Ratio

**Table 7:** Crude and adjusted association between inter-pregnancy interval and adverse maternal outcomes.

	Inter-pregnancy interval categories (months)				
	<24		24–59 (Ref.)	60+	
Outcome	OR (95%CI)	P-value		OR (95%CI)	P-value
Pre-eclampsia **(N = 7995)**	0.82 (0.62, 1.09)	0.174	1.00	1.05 (0.76, 1.44)	0.767
Pre-eclampsia^**a**^ **(N = 7749)**	**0.71 (0.52, 0.97)**	0.032^*^	1.00	0.92 (0.65, 1.27)	0.603
PPH **(N = 7360)**	0.82 (0.65, 1.05)	0.120	1.00	0.77 (0.58, 1.03)	0.081
PPH^**a**^ **(N = 7304))**	0.88 (0.68, 1.13)	0.311	1.00	**0.70 (0.52, 0.94)**	0.019^*^
Anemia **(N = 7995)**	0.82 (0.51, 1.31)	0.407	1.00	0.39 (0.18, 0.86)	**0.020**
Anemia^**a**^ **(N = 7906)**	0.72 (0.45, 1.17)	0.184	1.00	0.45 (0.20, 1.03)	0.059

^**a**^adjusted for: age, mother’s occupation, religion, current residence, alcohol use, pre-eclampsia in previous pregnancy, PPH in previous pregnancy, anemia in previous pregnancy, delivery mode, perinatal death of previous child at birth, parity, number of ANC visits, ANC attendance, type of family planning used, pregnancy type and referral status.

^*^Significant p-value

## Discussion

This study found that about half of the women at KCMC do not adhere to WHO recommendation on IPI. The median IPI increased yearly from 2002 to 2006, and then it plateaued and rose again in 2015.

We also noted that, IPI was shorter in younger women (<20 years) compared to older women and shorter in higher birth orders (seven and above) compared to lower birth orders. Our results suggest that women with short IPI are at lower risk of pre-eclampsia and those with long IPI are at lower risk of PPH. This implies sub-optimal IPI is protective for these adverse maternal outcomes. Lower risk of pre-eclampsia in short IPI was however incongruent with a study done in Latin America and The Caribbean[6], a difference which could probably be due to methodological differences. Conde-Agudelo’s [6] methodology ignored dependency which could have caused underestimation of standard errors, leading to smaller p-values and wrong inferences. In this study, data dependency was taken into consideration using robust standard errors, which gives our study greater strength. Although Conde-Agudelo [6] found no statistical significance in association of IPI on PPH in their study, the direction of effect of IPI was in agreement with this study.

Although the results in this study are inconsistent with other studies that precede it, we have reason to believe that the characteristics of women in this study could have contributed to and could explain the uncommon results reported in this study. KCMC is a zonal referral hospital and women delivering here are in the most part, high risk-pregnant women. Being a referral hospital, the health care workers are hyper vigilant and more equipped to address complications that could arise during pregnancy and child birth. This means that the type of care offered to high risk-pregnant women could be different from the type of care that these women could have received in lower level facilities and hence prevent these women from complications such as PPH ad Pre-eclampsia early on. This difference in care could be the reason why such associations are found to be protective.

The yearly increase in median IPI suggests an improvement during the early years of the study. This initial increase is likely to be influenced by increase in proportion of women using modern contraceptive, an almost constant total fertility rate from 1999 to 2005 and an increase in wanted fertility from 36% in 1999 to 42% in 2005 in Tanzania [31] The plateau from 2006 to 2014 implies changes in contraceptive use patterns. Although there was an increase in the proportion of women using modern contraceptives from 2005 to 2015, the greatest increase was noted in use of short term contraceptives [21] which are not as effective as long term contraceptives in affecting IPI[32]. This could explain the plateau. The rise seen in 2015 could suggest that, trends could be moving towards longer IPI. This could be due to re-launching of Green Star Campaign, a programmatic change made at the end of 2013 to promote the use of modern family planning methods, which could have influenced contraceptive use patterns[22]. The reported trend of median IPI from 2002 to 2015 in this study differs from that reported in the country [21] and this difference could be due to the fact that a survey reports a summary measure for all regions, therefore the regional differences are masked.

## Conclusion

There are still many women delivering within sub-optimal IPI (51.7%) in KCMC. Most women who have short IPI are young, less than 20years. This study has found a protective association between sub-optimal IPI with adverse maternal outcomes. However, these results do not nullify the previous literature as they could be biased by the demographics of these women as well as the tertiary care they are receiving
